# Supplementary material for: Comprehensive Proteome and Acetyl-Proteome Atlas Reveals Hepatic Lipid Metabolism in Layer Hens with Fatty Liver Hemorrhagic Syndrome
Source: Int J Mol Sci. 2023 May 9;24(10):8491. doi: 10.3390/ijms24108491 (PMC10217887; doi:10.3390/ijms24108491)
Supplement: Supplementary file 1 [file ijms-24-08491-s001.zip › ijms-2330460-supplementary/Supplementary figure S1.docx]

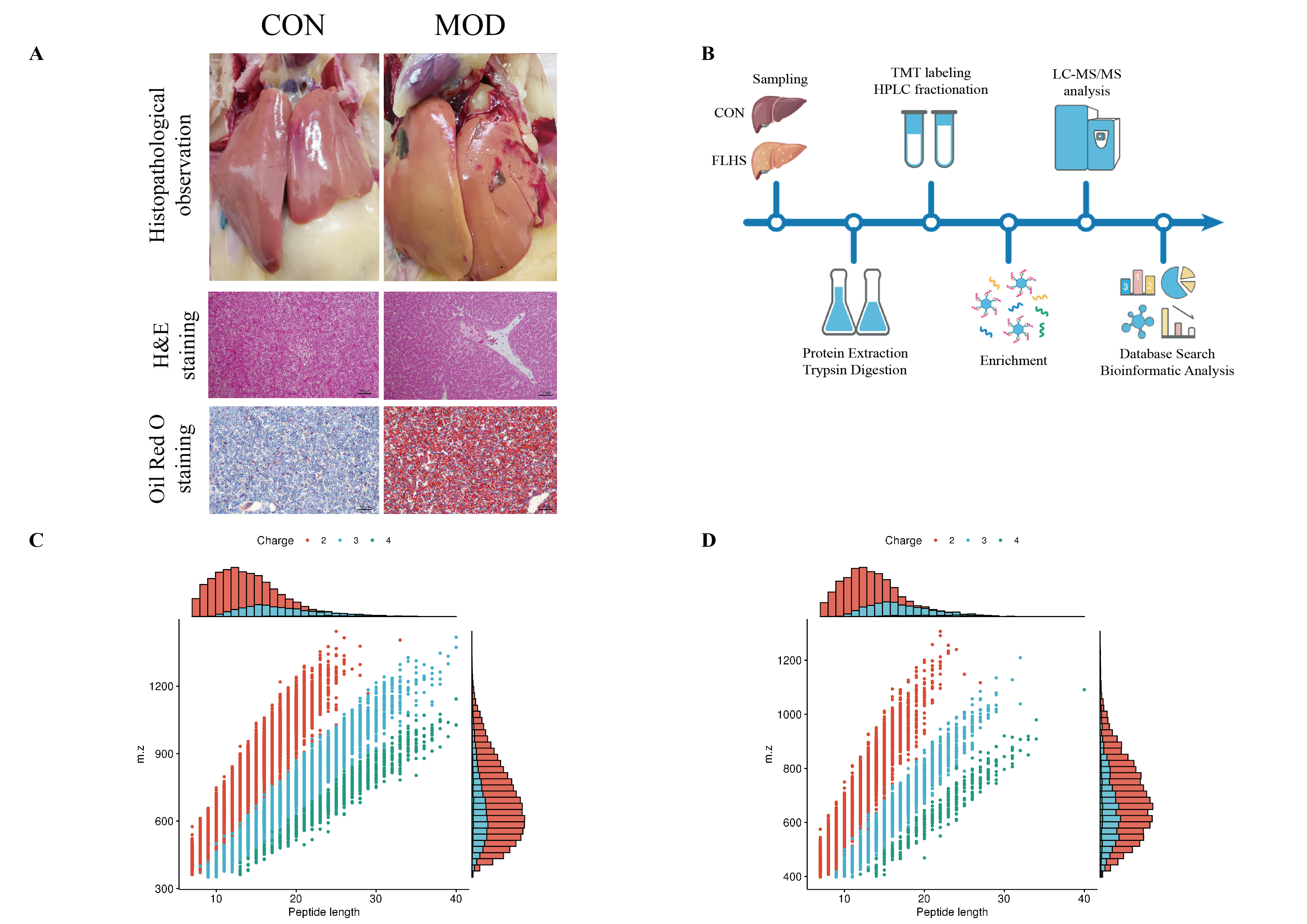


**Supplementary Figure S1.** Study design and data quality of proteome. (**A**) The histopathological observation, H＆E staining and oil red o staining of CON and MOD hens. (**B**) The whole experimental work-flow for the study. (**C**) The length of the peptide segments of SDEPs. (**D**) The length of the peptide segments of acetylated proteins.
